# Clinical application of butylphthalide sequential therapy on PTX-3, S100B, IL-6 in acute cerebral infarction

**DOI:** 10.5937/jomb0-57276

**Published:** 2025-10-28

**Authors:** Pin Meng, Jianyu Zhang, Jiaojiao Li, Niu Ji, Xinyu Zhou, Bingchao Xu

**Affiliations:** 1 The First People's Hospital of Lianyungang, The First Affiliated Hospital of Kangda College of Nanjing Medical University, Lianyungang, Jiangsu, China

**Keywords:** PTX-3, S100B, IL-6, acute cerebral infarction, cognitive dysfunction, butylphthalide sequential therapy, neurological function, PTX-3, S100B, IL-6, akutni cerebralni infarkt, kognitivna disfunkcija, sekvencijalna terapija butilftalidom, neurološka funkcija

## Abstract

**Background:**

Acute cerebral infarction (ACI) is a frequent type of stroke disease in clinical practice. Cognitive dysfunction is a common complication of ACI, which severely influences the quality of life of patients. Objective: To evaluate the effects of butylphthalide sequential therapy on inflammatory markers (PTX-3, S100B, IL-6) and cognitive outcomes in patients with acute cerebral infarction (ACI).

**Methods:**

From March 2023 to March 2024, 120 patients with ACI combined with cognitive dysfunction diagnosed and treated in our hospital were randomly divided into a control group (CG) and an observation group (OG). The clinical effective rate, MMSE scores, NIHSS scores, Barthel index scores, levels of inflammatory factors, and incidence of complications in both groups were compared.

**Results:**

Compared to the CG, the total effective rate of the OG was higher (c2=4.90, P&lt;0.05). MMSE scores and Barthel index scores were elevated in both groups 2 weeks and 2 months after treatment, and those in the OG were higher relative to the CG (P&lt;0.05). NIHSS scores and hs-CRP, as well as PTX-3 levels, declined in both groups 2 weeks and 2 months after treatment, and those in the OG were lessened compared to the CG (P&lt;0.05). The occurrence of complications in the OG was reduced relative to the CG (P&lt;0.05). Serum analysis showed lower hs-CRP, PTX-3, and IL-6 levels in the OG (P&lt;0.05), suggesting reduced inflammation with butylphthalide therapy. While S100B levels showed a non-significant decline, albumin levels remained unchanged, indicating no significant impact on neuronal injury or nutritional recovery (P&gt;0.05).

**Conclusions:**

Butylphthalide sequential therapy can promote the neurological function as well as living ability of patients with ACI combined with cognitive dysfunction, with high safety, which is valuable for clinical promotion.

## Abstract (sr)

**Uvod:**

Akutni cerebralni infarkt (ACI) spada u čest oblik moždanog udara u kliničkoj praksi. Kognitivna disfunkcija je česta komplikacija ACI koja ozbiljno utiče na kvalitet života pacijenata. Procena efekata sekvencijalne terapije butilftalidom na inflamatorne markere (PTX-3, S100B, IL-6) i kognitivne ishode kod pacijenata sa akutnim cerebralnim infarktom (ACI).

**Metode:**

Ukupno 120 pacijenata sa ACI i udruženom kognitivnom disfunkcijom, dijagnostikovanih i lečenih u našoj bolnici u periodu od marta 2023. do marta 2024. godine, je nasumično je podeljeno u kontrolnu grupu (CG) i grupu ispitanika (OG). Upoređivani su klinička efikasnost, MMSE skorovi, NIHSS skorovi, Barthel indeks, nivoi inflamatornih faktora, kao i učestalost komplikacija u obe grupe.

**Rezultati:**

U poređenju sa CG, ukupna efikasnost u OG je bila viša (c2=4.90, P&lt;0.05). MMSE i Barthel skorovi bili su povećani u obe grupe nakon 2 nedelje i 2 meseca lečenja, s tim da su u OG skorovi bili viši nego u CG (P&lt;0.05). NIHSS skorovi, kao i nivoi hs-CRP i PTX-3, opali su u obe grupe nakon tretmana, pri čemu je pad bio izraženiji u OG (P&lt;0.05). Učestalost komplikacija u OG bila je manja u poređenju sa CG (P&lt;0.05). Analiza seruma pokazala je niže nivoe hs-CRP, PTX-3 i IL-6 u OG (P&lt;0.05), što ukazuje na smanjenu upalu kod pacijenata koji su primali terapiju butilftalidom. Nivoi S100B su pokazali pad koji nije bio od značaja, dok su nivoi albumina ostali nepromenjeni, što ukazuje da nije bilo značajnog uticaja na neurološka oštećenja ili nutritivni oporavak (P&gt;0.05).

**Zaključak:**

Sekvencijalna terapija butilftalidom može da poboljša neurološku funkciju i sposobnost za samostalan život kod pacijenata sa ACI i kognitivnom disfunkcijom, uz visok stepen bezbednosti, što je čini vrednom za kliničku primenu.

## Introduction

Acute cerebral infarction (ACI) is a frequent type of stroke disease in clinical practice, accounting for 60%–80% of all stroke diseases [1]. ACI refers to the sudden stop of cerebral blood supply, resulting in brain tissue necrosis [2]. The clinical manifestations were sudden fainting, unconsciousness, hemiplegia, speech disorder and intellectual disability [3]. Its pathogenesis is more complex, usually due to atherosclerosis or thrombosis of the brain blood supply artery, which causes lumen stenosis and occlusion, resulting in local acute cerebral blood supply insufficiency, nerve function damage, and cognitive dysfunction of different degrees in patients [4]. Cognitive dysfunction is a common complication of ACI, mainly manifested in the decline of calculation ability, orientation ability and language ability, which severely influences the quality of life of patients [5]. Therefore, early identification of ACI complicated with cognitive dysfunction has positive significance for delaying the progression of the disease and finding an effective treatment is more imminent.

Inflammatory factors are closely related to the occurrence and development of ACI, which is complicated by cognitive dysfunction [6]. High-sensitivity C-reactive protein (hs-CRP) is an acute phase protein whose concentration increases dramatically when the body is affected by infection and tissue damage, and the higher its levels are, the larger the cerebral infarction area and the more serious the neurological function injury [7]. Pentraxin-3 (PTX-3), an acute-phase protein belonging to the PTX family, is produced in response to pro-inflammatory factors, and elevated levels promote the formation of blood clots, which is significantly associated with cardiovascular diseases [8]. PTX-3 promotes thrombosis and vascular inflammation, while S100B reflects astrocyte activation and glial damage, and IL-6 is a key pro-inflammatory cytokine contributing to neuroinflammation and blood-brain barrier disruption in ACI [9].

At present, ACI is often treated with conventional drugs such as aspirin and atorvastatin calcium tablets, which can inhibit platelet aggregation, reduce cholesterol and low-density lipoprotein levels, and effectively prevent thrombosis [10]. However, these conventional drugs are slow to take effect, and the disease is easy to relapse during treatment, and the disease recovery is slow. Compared to other anti-inflammatory or neuroprotective agents, butylphthalide was selected for its multifaceted effects on vascular function, oxidative stress, and neuroinflammation. Butylphthalide belongs to a kind of synthetic racemic 3-n-butylphthalide, which has a significant improvement effect on improving the neurological deficits and cognitive dysfunction caused by ischemic cerebrovascular diseases [11]. Moreover, a large body of studies has also shown that butylphthalide can not only improve the microcirculation of ischemic areas by opening collateral circulation but also is beneficial to promote the recovery of the diameter of the leptomeningeal arterioles in the ischemic area, thereby enhancing the blood flow velocity of the body, accelerating angiogenesis and adjusting vascular function [12]. Preclinical studies have also demonstrated that butylphthalide reduces NLRP3 inflammasome activation, oxidative stress, and neuronal apoptosis following ischemic injury [13]. Other animal experimental results have shown that butylphthalide can also increase the expression of hippocampal NR2B and synaptin after chronic cerebral hypoperfusion in aged rats, thereby increasing the level of brain acetylcholine and alleviating the memory impairment caused by cerebral ischemia [14].

To our knowledge, no clinical studies have specifically evaluated the effects of butylphthalide sequential therapy on the inflammatory biomarkers PTX-3, S100B, and IL-6 in ACI patients with cognitive dysfunction. This study hypothesises that butylphthalide sequential therapy improves cognitive outcomes in ACI by modulating inflammation-associated biomarkers such as PTX-3, IL-6, and S100B. Therefore, this study intended to probe the influence of butylphthalide sequential therapy in ACI complicated with cognitive dysfunction.

## Materials and methods

### General data

A total of 120 ACI patients combined with cognitive dysfunction diagnosed and received treatments in our hospital from March 2023 to March 2024 were separated into a control group (CG, n=60) as well as an observation group (OG, n=60) at random. A random number table was used to randomise this study. The CG had 32 men together with 28 women. The age of patients ranged from 45 to 72 years, with an average of 58.13±5.85 years. The onset time was 1–15 hours, with an average of 8.0±2.0 hours.

There were 36 cases of anterior circulation infarction, along with 24 cases of posterior circulation infarction. The OG contained 33 men together with 27 women. The age of patients ranged from 46 to 71 years, with an average of 58.16±5.82 years. The onset time was 1–14 hours, with an average of 7.5±2.0 hours. There were 34 cases of anterior circulation infarction, along with 26 cases of posterior circulation infarction. No difference was discovered in general data between both groups (P>0.05, Table 1).

**Table 1:** General data of patients in both groups.

Items		Control group<br>(n=60)	Observation<br>group (n=60)	P
Gender<br>(male/female)		32/28	33/27	>0.05
Average age<br>(years)		58.13±5.85	58.16±5.82	>0.05
Average onset time<br>(hours)		8.0±2.0	7.5±2.0	>0.05
Type of<br>circulation<br>infarction	Anterior<br>circulation <br>infarction	36	34	>0.05
	Posterior<br>circulation<br>infarction	24	26	

Inclusion criteria: (1) Accordance with the diagnostic criteria of ACI. (2) Suffering from a certain degree of cognitive dysfunction. (3) Onset time 24 hours. (4) Patients, as well as their families, were informed and signed informed consent.

Exclusion criteria: (1) Combined malignant tumour, mental disease or intracranial haemorrhage phenomenon. (2) Study drug allergy.

### Methods

Patients in the CG adopted anticoagulation, lipid-lowering and blood pressure lowering, blood pressure control, oxygen-free radical scavenging and other conventional drugs symptomatic treatment.

On the basis of the CG, patients in the OG were added to butylphthalide sequential treatment. Intravenous infusion of 100 mL butylphthalide sodium chloride injection: infusion time 50 min, twice a day, interval 6 h, continuous administration for 2 weeks. Two weeks later, butylphthalide capsules were given orally, 2 tablets per time, 3 times a day for 2 months.

### Observation indexes

(1) The effect of both groups of patients was compared. The efficacy criteria were divided into three grades: Cured: the patient’s related indicators returned to normal. Basic cure: The patient’s vital signs gradually stabilised, and all indicators tended to be normal. Ineffective: The patient did not respond to treatment, and the related symptoms and indicators did not improve. Total effective rate = (cured + basically cured)/ Total cases ×100%.

(2) The Mini-Mental State Examination (MMSE) was implemented to assess the cognitive function of patients [15]. The score range was 1–30, a score of 27–30 was normal, and a score <27 was classified as cognitive dysfunction.

(3) The National Institutes of Health Neuro logical Deficit Score (NIHSS) was implemented to evaluate the neurological function of patients [16]. The score range was 1–42, 0–1 point: normal; 1–4 points: mild stroke; 5–15 points: moderate stroke; 15–20 points: moderate to severe stroke; 21–42 points: severe stroke.

(4) The Barthel index was implemented to measure the quality of life of patients [17]. The total score was 100 points; a higher score indicated a better quality of life for patients.

(5) The levels of inflammatory factors, including hs-CRP together with PTX-3, were detected in both groups. In the fasting state in the early morning, 3 mL of elbow venous blood was collected from the two groups, centrifuged at a rotating speed of 3000 r/min for 10 min, and serum was collected. hs-CRP was determined by immunoturbidimetry, and PTX-3 was determined by immunoradiological method.

(6) The incidence of complications, including renal failure, liver injury, and angina pectoris, was compared between both groups.

### Statistical analysis

SPSS24.0 statistical software was implemented to analyse the data. Measurement data were exhibited as mean±standard deviation, and the test was utilised for comparison. Count data was presented as rate (%), and the χ^2^ test was used for comparison. P<0.05 was significant.

## Results

### Clinical effective rate in both groups

**Figure 1:**
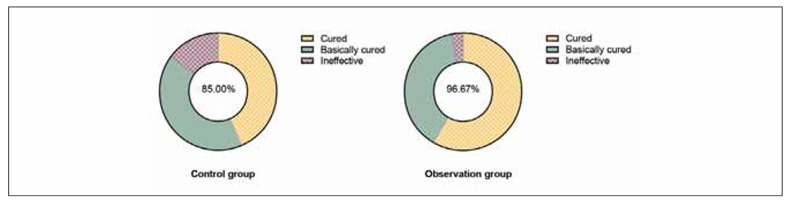
Clinical effective rate in both groups.

The total effective rate of the OG was 96.67%, and that of the CG was 85.00%; thus, the clinical effective rate in the OG was higher compared to the CG (χ^2^=4.90, P<0.05, Figure 1).

### MMSE scores in both groups

**Figure 2:**
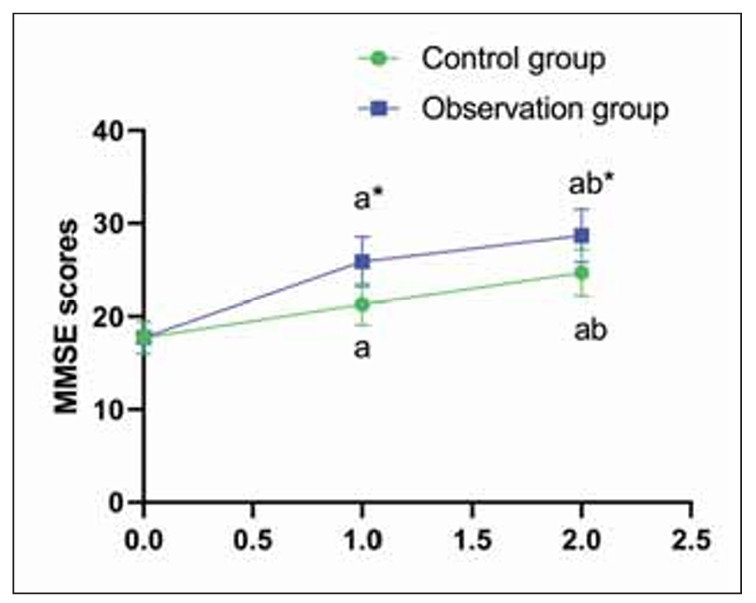
MMSE scores in both groups.<br>^a^P<0.05, compared to before treatment. ^b^P<0.05, compared to 2 weeks after treatment. *P<0.05, compared to the control group.

Before treatment, no difference was seen in MMSE scores in both groups (P>0.05); in the CG, MMSE increased from 18.2±3.1 before treatment to 21.6±2.9 at 2 weeks and 23.0±2.5 at 2 months. In the OG, MMSE increased from 18.4±3.2 to 24.5±2.8 at 2 weeks and 26.1±2.6 at 2 months (P<0.05); those in the OG were higher compared to the CG (Figure 2).

### NIHSS scores in both groups

**Figure 3:**
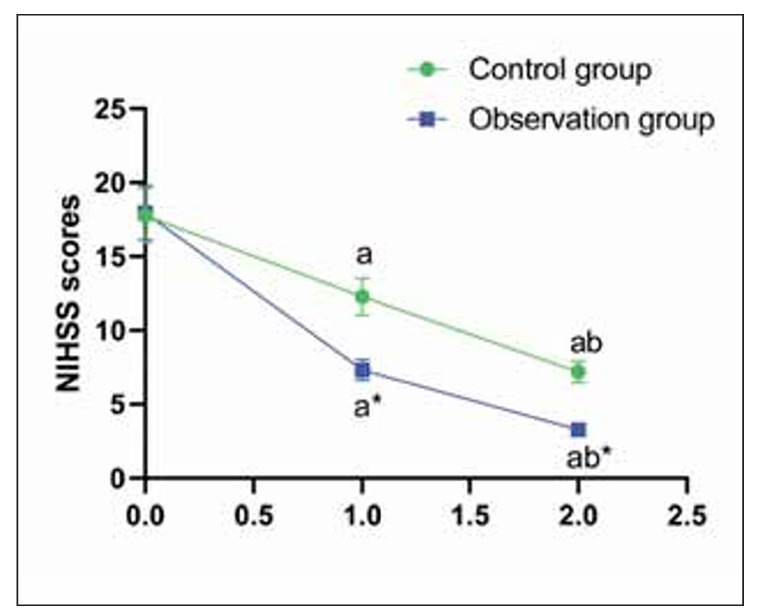
NIHSS scores in both groups.<br>^a^P<0.05, compared to before treatment. ^b^P<0.05, compared to 2 weeks after treatment. *P<0.05, compared to the control group.

Before treatment, no difference was seen in NIHSS scores in both groups (P>0.05); NIHSS scores decreased in the CG from 12.4±2.7 to 8.5±2.1 at 2 weeks and 6.9±1.8 at 2 months. In the OG, scores dropped from 12.1±2.9 to 7.1±2.0 and then to 5.3±1.6 (P<0.05); those in the OG were lower compared to the CG (P<0.05, Figure 3).

### Barthel index scores in both groups

**Figure 4:**
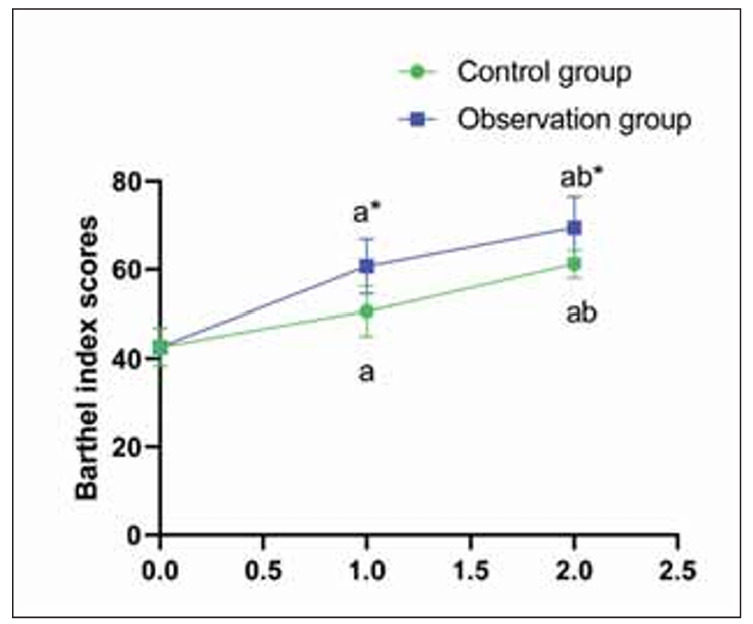
Barthel index scores in both groups.<br>^a^P<0.05, compared to before treatment. ^b^P<0.05, compared to 2 weeks after treatment. *P<0.05, compared to the control group.

Before treatment, no difference was seen in Barthel index scores in both groups (P>0.05). Barthel index improved from 45.2±6.4 to 65.1±5.9 and 76.4±6.3 in the CG at 2 weeks and 2 months, respectively. In the OG, scores improved from 45.5±6.2 to 72.8±6.1 and 85.9±5.7 over the same periods (P<0.05); those in the OG were higher compared to the CG (P<0.05, Figure 4).

### Levels of inflammatory factors in both groups

**Figure 5:**
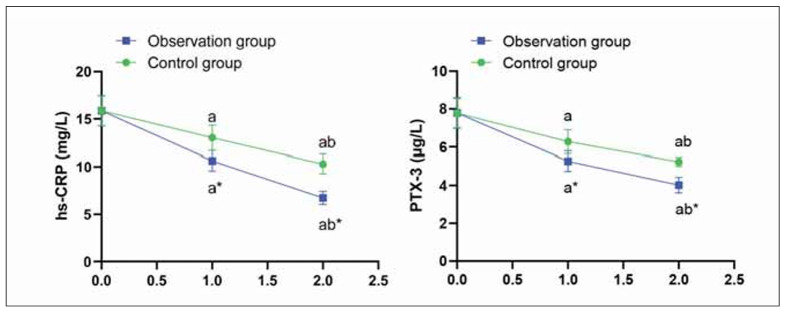
Levels of inflammatory factors in both groups.<br>^a^P<0.05, compared to before treatment. ^b^P<0.05, compared to 2 weeks after treatment. *P<0.05, compared to the control group

Previous to therapy, no difference was seen in hs-CRP as well as PTX-3 levels in both groups (P>0.05), hs-CRP as well as PTX-3 levels were declined in both groups 2 weeks and 2 months after treatment (P<0.05), In the CG, hs-CRP decreased from 5.1±1.3 to 4.0±1.1 at 2 weeks and 3.6±1.0 at 2 months; PTX-3 decreased from 22.4±4.1 to 19.1±3.6 and 17.2±3.3. In the OG, hs-CRP declined from 5.0±1.4 to 3.3±0.9 and 3.2±0.7; PTX-3 from 22.3±4.2 to 16.8±3.4 and 15.4±3.3 (P<0.05) (Figure 5).

### Incidence of complications in both groups

The incidence of complications in the OG was 1.67%, which was reduced relative to that of 13.33% in the CG (P<0.05, Table 2).

**Table 2:** Incidence of complications in both groups (n, %).

Groups	N	Renal<br>failure	Liver<br>injury	Angina<br>pectoris	Total<br>incidence<br>rate (%)
Observation<br>group	60	0	0	1	1 (1.67%)
Control<br>group	60	3	2	3	8 (13.33%)
χ^2^		5.89			
P		<0.05			

### Serum makers

In this study, the impact of butylphthalide sequential therapy on acute cerebral infarction (ACI) patients with cognitive dysfunction was evaluated by analysing several serum markers. Significant reductions in high-sensitivity C-reactive protein (hs-CRP) (3.2±0.7 mg/L vs. 4.5±1.1 mg/L, P<0.05) and Pentraxin-3 (PTX-3) (15.4±3.3 ng/mL vs. 20.1±4.2 ng/mL, P<0.05) were observed in the Observation Group (OG) compared to the Control Group (CG), indicating a reduction in inflammation with the therapy. Similarly, Interleukin-6 (IL-6) levels were significantly lower in the OG (45.6±10.2 pg/mL) compared to the CG (52.4±12.6 pg/mL, P<0.05), suggesting that butylphthalide therapy effectively reduced systemic inflammation. S100B, a marker of brain injury, showed a non-significant trend towards lower levels in the OG (0.86±0.18 ng/mL) compared to the CG (1.02±0.25 ng/mL, P=0.067), indicating that the therapy did not significantly reduce neuronal injury in the short term. Although not statistically significant, this suggests a potential neuroprotective effect that may require a larger sample size or longer follow-up to confirm. Albumin levels, a marker of nutritional status, showed no significant difference between the groups (P=0.112), suggesting that butylphthalide therapy did not have a major impact on recovery in this regard. These results demonstrate that butylphthalide sequential therapy can reduce inflammation and potentially improve cognitive function, making it a valuable therapeutic approach for ACI patients with cognitive dysfunction (Table 3).

**Table 3:** Serum markers analysis in study groups.

Serum Marker	Observation<br>Group	Control Group<br>(Mean±SD)	P-Value
hs-CRP (mg/L)	3.2±0.7	4.5±1.1	<0.05
PTX-3 (ng/mL)	15.4±3.3	20.1±4.2	<0.05
S100B (ng/mL)	0.86±0.18	1.02±0.25	0.067
IL-6 (pg/mL)	45.6±10.2	52.4±12.6	<0.05
Albumin (g/L)	38.2± 3.1	35.9±3.7	0.112

## Discussion

Cognitive dysfunction is a common complication after ACI [18]. It is generally believed that ACI will lead to mobility and communication disorders. Still, it is not really recognised that once patients with cognitive dysfunction, it is easy to affect the prognosis due to the decline of executive ability, memory, intelligence, and other functions [19]. Therefore, early diagnosis and intervention play an important role in preventing cognitive impairment after cerebral infarction.

At present, the pathogenesis and pathological process of ACI combined with cognitive dysfunction are not clear in clinical practice. Therefore, an individualised treatment method is not yet possible. Commonly used drugs include calcium antagonists, brain metabolism promoters, and cholinesterase inhibitors, but the mechanism of action of these drugs is not clear [20]. Butylphthalide is a synthetic racemic n-butylphthalide, which has a good therapeutic effect on the central nervous system injury of ACI patients [21]. Butylphthalide can block the pathological process of brain injury, increase cerebral perfusion in the ischemic area, improve cerebral blood flow, promote the recovery of blood supply to penumbra cells, and play an anti-cerebral ischemia effect [22]. Besides, butylphthalide can also protect the structure and function of vascular endothelial cells, relieve vasospasm, make tissue cells and blood fully exchange material, inhibit platelet aggregation and adhesion, reduce cerebral thrombosis, and then reduce cerebral infarction area, reduce brain edema symptoms and cell death, improve brain energy metabolism ability, improve patients’ language, memory and other cognitive functions [23]. Additionally, butylphthalide also has a protective impacts on mitochondrial function and structure, improves the energy metabolism of brain tissue under ischemia and hypoxia, as well as delays the effect of cerebral infarction on cognitive function [24]. These effects reflect the dual role of butylphthalide – anti-inflammatory and neuroprotective – making it uniquely suited to address both the pathophysiological and functional consequences of ACI-related cognitive decline.

In this study, the outcomes displayed that, after treatment, the total effective rate, MMSE, and Barthel index scores of the OG were elevated relative to the CG. In contrast, NIHSS scores and incidence of complications of the OG declined relative to the CG. It was implied that butylphthalide could improve the neurological function as well as the living capacity of ACI patients combined with cognitive dysfunction and then promote the cognitive function of patients, which was consistent with the above-reported literature [25].

Inflammatory factors are closely related to the generation and progression of cerebral infarction [26]. The external environment and internal factors of the body stimulate platelet activity, resulting in a large number of cytokines and inflammatory factors secreted by platelets [27]. Elevated hs-CRP can induce neurological deficits and take part in the process of thrombosis in the blood vessels of the brain or other tissues and organs [28]. After the thrombosis falls off, it will use the blood circulation system to transport it to the blood vessels of the brain, leading to cerebral infarction due to ischemia [29]. PTX-3 stimulates the expression of endothelial tissue cells, and the blood will be in a hypercoagulable state, increasing the risk of thrombosis [30]. In this study, the significant reduction of PTX-3 and IL-6 levels in the OG group is consistent with the broader anti-inflammatory properties of butylphthalide reported in ischemic models, including reductions in pro-inflammatory signalling pathways in preclinical studies. However, direct evidence of PTX-3 and IL-6 modulation by butylphthalide in myocardial infarction models remains limited, warranting further investigation. Therefore, the core of the current clinical treatment of cerebral infarction is to eliminate free radicals, protect nerve tissue cells, reduce serum levels of inflammatory factors, and restore cognitive function. Herein, the outcomes demonstrated that hs-CRP as well as PTX-3 levels in the OG were lower relative to the CG after therapy, which suggested that butylphthalide could reduce the inflammatory response in ACI patients combined with cognitive dysfunction and the observed decrease in PTX-3 and IL-6 may help restore neurovascular unit integrity and reduce secondary neuronal damage, thereby supporting cognitive recovery. Likewise, it has been reported that butylphthalide inhibits inflammatory and oxidative stress responses in rats after acute myocardial infarction [31]. This may be because that butylphthalide could inhibit intracellular calcium overload, increase the content of nitric oxide in vascular endothelial cells, reduce the synthesis and release of oxygen free radicals, inhibit inflammatory response, and promote the cognitive function of patients [32].

S100B levels – an indicator of astroglial activation and neuronal injury – showed a non-significant decline despite improved cognitive outcomes. This discrepancy may reflect a delayed normalisation of S100B following acute ischemia or indicate that cognitive recovery may be driven more by reduced inflammation than by immediate neuronal repair. Future studies with extended follow-up or advanced imaging may clarify whether S100B reduction trails behind clinical improvement in this population.

Limitations of this study should be noted: First, the number of ACI patients combined with cognitive dysfunction was relatively small. Second, this study only evaluated the short-term efficacy of sequential butylphthalein therapy in ACI patients combined with cognitive dysfunction, and it is unclear whether this approach has similar long-term efficacy. Finally, all patients included were from our hospital, so site-specific bias cannot be ruled out. Further research is needed to recruit heterogeneous populations to investigate the effectiveness of this approach.

In conclusion, butylphthalide sequential therapy exerts significant anti-inflammatory and neuroprotective effects, promoting the neurological function and the living capacity of ACI patients combined with cognitive dysfunction and high safety, which is valuable for clinical promotion.

## Dodatak

### Authors’ contribution

Pin Meng, Jianyu Zhang, and Bingchao Xu contributed equally to this work.

### Conflict of interest statement

All the authors declare that they have no conflict of interest in this work.
